# Brazilian pregnant and lactating women do not change their food intake to meet nutritional goals

**DOI:** 10.1186/1471-2393-14-186

**Published:** 2014-06-02

**Authors:** Quenia dos Santos, Rosely Sichieri, Dirce ML Marchioni, Eliseu Verly Junior

**Affiliations:** 1 Institute of Social Medicine, State University of Rio de Janeiro, Rua São Francisco Xavier, 524, Maracanã, Rio de Janeiro 20550-900, Brazil; 2 Department of Nutrition, School of Public Health, University of São Paulo, Av Doutor Arnaldo 715, São Paulo 01246-904, Brazil

**Keywords:** Pregnancy, Micronutrients, Maternal nutrition, Lactation, Food intake

## Abstract

**Background:**

Nutritional requirements are increased during pregnancy and lactation. The aim of this study was to compare the food intake and prevalence of inadequate nutrient intake among pregnant, lactating and reproductive-age women.

**Methods:**

Two-day dietary records of 322 pregnant and 751 lactating women were compared to those of 6837 non-pregnant and non-lactating women aged 19 to 40 years from a nationwide representative sample. The usual nutrient intake was estimated using the National Cancer Institute method, and compared to nutritional goals to estimate prevalence of inadequate intake.

**Results:**

Pregnant, lactating and reproductive-age women did not differ in their average consumption of 18 food groups, except for rice, with greatest intake among lactating women. The prevalence of nutrient inadequacy in pregnant women was higher than in reproductive-age women for folate (78% versus 40%) and vitamin B6 (59% versus 33%). In lactating women, prevalence was higher than in reproductive-age women for vitamin A (95% versus 72%), vitamin C (56% versus 37%), vitamin B6 (75% vs. 33%), folate (72% versus 40%) and zinc (64% versus 20%). The percentage of sodium intake above the upper limit was greater than 70% in the three groups.

**Conclusions:**

Inadequate intake is frequent in women and increases during pregnancy and lactation, because women do not change their food intake. Guidelines should stimulate healthy food intake for women across the lifespan.

## Background

Pregnancy and lactation increase nutritional requirements. This higher demand of energy and nutrients is necessary to support the growth and development of the foetus and child and associated changes in maternal metabolism [1].

Poor diet during pregnancy has been associated with adverse outcomes, such as abnormal foetal growth [2,3] birth defects [4] and increased risk of hypertensive disorders [5,6]. Specific micronutrient deficiencies can result in a low birth weight, and maternal obesity is associated with the development of gestational diabetes and/or hypertension syndrome during pregnancy, which can have consequences for the health of both the mother and the newborn [7].

For lactating women, the nutritional demands are considerably greater than those of pregnancy, and diet can affect the synthesis, composition, and secretion of milk [8,9]. In the postpartum period, mothers are generally advised to increase their energy intake to meet the cost of lactation. However, considering that obesity and being overweight are major problems among Brazilian women [10] and worldwide and because parity is associated with obesity [8], increasing the quality of the diet and reducing excessive energy intake during pregnancy should be a key message.

Pregnant and lactating women should have particular concerns about their food choices compared to other women in order to support their higher nutritional requirements. The aim of this study is to compare food and identify the prevalence of inadequate nutrient intake amongst pregnant, lactating and reproductive-age women.

## Methods

### Study population

The study analysed data from the first National Dietary Survey (NDS) [11], which was conducted along with the Household Budget Survey (HBS) 2008–2009 carried out by the Brazilian Institute of Geography and Statistics.

Briefly, HBS 2008–2009 adopted a two-stage cluster sampling. In the first stage, primary sampling units (PSU) were selected by systematic sampling with probability proportional to the number of households according to the 2000 Census. For the systematic sampling, PSUs were stratified according to main geographical areas, urban or rural situation, and the mean income of heads of households. The units sampled in the second stage of selection were the permanent households selected by simple random sampling without replacement within each sector. PSUs were evaluated throughout 12 months of research in all strata of the research [11].

A subsample for the NDS was set at 25% of the households sampled for the HBS 2008–2009, which were further selected by simple random sampling, resulting in a total of 16,764 households invited, of which 13,569 households responded to the survey. The final sample was composed of 33,004 individuals ten years and older.

Details on sample data collection are available elsewhere [11,12]. Our analysis included data on food consumption of 322 pregnant and 751 lactating women in Brazil, aged 19–40 years, compared to all non-pregnant and non-lactating women within the same age range (n = 6837).

### Assessment of food intake

Dietary intake was collected from two non-consecutive food records. Individuals recorded all foods and beverages consumed during one day, including the time of intake, quantities consumed in portion sizes, preparation form, and the location (inside or outside the home). Additionally, a question related to the consumption of sugar and sweetener was included. In order to guide accurate recording, each participant received instructional material with guidance on filling in the food record and photographs of utensils commonly used to serve the foods and drinks. When the interviewee was unable to complete the food records, or when the interviewee was illiterate, someone indicated by the interviewee completed the record.

Quality of the records was ensured through checking by research assistants (RAs) at the interviewee´s house, using methods already tested in other studies [13,14]. They included:

1. When there was no record of any food in an interval of at least three hours, the RAs were instructed to confirm whether the respondent actually did not consume any product in that period;

2. When less than five items were recorded over a day, RAs asked residents if other foods were consumed that may have been forgotten;

3. The research assistants were also instructed to inquire whether foods usually omitted in dietary surveys had been consumed, such as small snacks, candies, pastries, coffee, sodas, and other beverages. In addition, research assistants checked if reduced energy products or diet products were consumed.

All of the information was entered into a laptop computer in the household, using a program specifically designed for data entry on food consumption. The database comprised approximately 1,500 items (food and beverages) that were selected from the data acquisition of food and drinks of HBS 2002–2003. Foods that were not included on this list could be added at any time.

The addition of soya oil to all meat, fish and poultry dishes and to boiled and sautéed vegetables was taken into account. Moreover, the addition of 10 g of sugar (standardized) to every 100 ml of fruit juice, coffee, coffee and milk, tea and mate (a typical Brazilian tea) was taken into account when the subject reported using sugar as usual and 5 g of sugar for every 100 ml when the subject reported using both sugar and sweetener [15].

To calculate the nutritional value of each food consumed, the Brazilian Table of Food Composition (TACO) and the Nutrient Data System for Research were used. The nutrient composition and portion sizes, which were specifically compiled for the analysis of foods and preparations cited on the HBS 2008–2009, were selected from the 5,686 items registered in the food and drink database from the HBS 2002–2003.

Partial analyses were performed during the data collection to monitor quality control, frequency response, the average items consumed in the first and second days of the food records, coding of unregistered items, and item analyses which were improperly included. In addition, quantities considered to be unlikely were entered using the hot deck imputation procedure and this information was registered in the database.

Details of the pre-test, training and validation of the data collection can be found elsewhere [12].

### Data analysis

The distribution of intake based on only two days is subject to day-to-day fluctuations (within-person variation), which may under- or overstate the prevalence of inadequacy [16]. The method of the National Cancer Institute (NCI) [12,17] was used to remove thse within-person variance allowing estimation of their usual dietary intake. Macros developed by the NCI in Statistical Analysis System (SAS) are available in [18].

This method consists of a two-part nonlinear mixed model. The first part of the model estimates the probability of consumption using logistic regression, adjusting for the person-specific random effect; the second part specifies the consumption-day amount using linear regression on a transformed scale, also accounting for the person-specific effect. The person-specific random effects were allowed to be correlated across the two parts of the model because the probability of consumption is often related to the amount consumed. For foods that are assumed to be episodically consumed the two part model was used. For nutrients that are assumed to be ubiquitously consumed the probability part of the model was not needed, and the one-part model was used. For these analyses, the covariates were as follows: 1) dummy variables indicating pregnancy and lactation, 2) urban or rural area and 3) regions of the country.

The EAR (Estimated Average Requirement) cut-point method was used to calculate the prevalence of inadequate intakes of calcium, zinc, folate, vitamin A, vitamin B6, vitamin B12 and vitamin C [19,20]. This method assumes that the requirement of symmetry is met, which was the case for all nutrients, except iron in reproductive-age women, because iron is lost during menstruation [21]. In this case, we used the probabilistic approach [22].

Sodium intake was assessed with respect to the Tolerable Upper Intake Level (UL), which estimates the percentage of a population potentially at risk for adverse effects [23]. This measurement accounts for both the intrinsic sodium content in food and the sodium added to it.

The 95% confidence intervals of the means and proportions were calculated from the standard errors, estimated by the Balanced Repeated Replication technique [12,24]. Standard errors for iron were not calculated. All analyses were performed using SAS software (Statistical Analysis System), version 9.1. Significant differences among the groups were evaluated by no- intersection among 95% confidence intervals.

Socio-economic variables were obtained by personal interview. Schooling was defined by the number of completed years of study. We grouped women who reported that they did not know how many years they went to school into the category "less than one year of study". Total income was obtained through the sum of the gross cash income of the residents of the house, excepting domestic employees and their relatives, plus the total non-monetary income. The total per capita family income was categorised according to the minimum wage from January 15, 2009 [11]. This study was approved by the ethics committee of the Institute of Social Medicine of State University of Rio de Janeiro (CAAE 0011.0.259.000-11).

## Results

The mean age, schooling and income of the three groups are shown in Table 1. Pregnant women are younger and have fewer years of education compared to reproductive-age women (8.7 versus 10.2); whereas, lactating women reported lower incomes compared to the other two groups.

**Table 1:** Characteristics of the study population

	**Pregnant women (n = 322)**				**Lactating women (n = 751)**				**Reproductive-age women (n = 6837)**			
	**P5**	**Mean**	**95% CI**	**P95**	**P5**	**Mean**	**95% CI**	**P95**	**P5**	**Mean**	**95% CI**	**P95**
**Age**	19.0	26.6	(25.9; 27.3)	37.0	20.0	27.6	(26.8; 28.3)	37.0	20.0	29.4	(29.2; 29.7)	39.0
Schooling^*^	2.0	8.7	(8.4; 9.0)	15.0	1.0	9.1	(8.0, 10.1)	15.0	2.0	10.2	(9.8; 10.6)	15.0
**Income**	68.2	1.7	(1.5; 1.8)	1734.8	52.3	1.0	(0.9; 1.1)	1272.5	90.3	1.9	(1.8; 2.0)	2078.0

*Schooling in years of study.

Household income based on minimum salary on January 15,2009 (1 salary = US$ = 180,00).

The mean energy intake of pregnant women was 1964 kcal (95% CI = 1720; 2208); for lactating women it was 1804 kcal (95% CI = 1658; 1950) and for reproductive age-women it was 1757 kcal (95% CI = 1744; 1770). There were no differences in energy intake between the groups.

Regarding the consumption of food items (Table 2), except for rice in lactating women, there were no differences between pregnant, lactating or reproductive-age women.

**Table 2:** Average intake (g) of food items and 95% Confidence Interval (95% CI) based on two days of dietary records

	**Pregnant women**		**Lactating women**		**Reproductive-age women**	
**Food item**	**Average intake (g)**	**95% CI**	**Average intake (g)**	**95% CI**	**Average intake (g)**	**95% CI**
Rice	152.1	(128.4; 175.8)	164.8	(151.5; 178.1)	145.1	(141.2; 149.0)
Beans	167.6	(145.1; 190.1)	183.2	(170.1; 196.3)	166.4	(157.2; 175.2)
Corn and corn based preparations	21.7	(17.6; 25.8)	21.7	(16.4; 26.9)	18.1	(15.5; 20.6)
Pasta	44.1	(38.2; 49.9)	40.9	(26.2; 55.6)	48.1	(45.9; 50.2)
Eggs	10.3	(9.3; 11.3)	10.5	(9.7; 11.3)	10.4	(9.8; 10.9)
Meats	153.2	(136.9; 169.4)	141.8	(124.1; 159.4)	132.9	(125.8; 139.9)
Processed meats	7.5	(4.9; 10.0)	8.0	(7.0; 8.9)	7.2	(5.8; 8.6)
Breads	50.0	(40.0; 59.9)	52.7	(48.4; 57.0)	47.2	(43.1; 51.3)
Candies and cakes	41.4	(34.9; 47.9)	45.5	(39.0; 51.9)	38.1	(34.9; 41.2)
Cookies	12.2	(9.3; 15.1)	12.1	(10.7; 13.5)	12.0	(10.2; 13.8)
Pizzas, snacks and sandwiches	30.6	(23.9; 37.3)	27.8	(24.5; 31.1)	27.7	(26.7; 28.7)
Greens and vegetables	46.1	(39.2; 52.9)	40.2	(36.9; 43.5)	39.2	(36.6; 41.7)
Fruits	81.0	(63.5; 98.4)	86.5	(73.6; 99.4)	79.7	(73.6; 85.8)
Oils and fats	6.3	(5.3; 7.3)	6.0	(4.2; 7.7)	5.7	(5.1; 6.3)
Milk and dairy produtcs	93.4	(71.5; 115.3)	75.9	(62.6; 89.3)	68.7	(64.8; 72.5)
Juices	104.9	(82.2; 115.3)	118.4	(110.2; 126.6)	107.7	(99.1; 116.3)
Coffee	155.6	(73.5; 237.7)	213.8	(193.8; 233.8)	194.7	(183.3; 206.1)
Soft drinks	92.6	(83.9; 101.2)	93.7	(87.2; 100.2)	93.5	(88.0; 98.9)

The prevalence of inadequate nutrient intake was higher in pregnant women than in reproductive-age women (Table 3) for folate (78% versus 40%) and vitamin B6 (59% versus 33%); and in lactating women compared to reproductive-age women for vitamin A (95% versus 72%), vitamin C (56% versus 37%), vitamin B6 (75% vs. 33%), folate (72% versus 40%) and zinc (64% versus 20%).

**Table 3:** EAR, average intake and% of inadequacy (with their respective 95% Confidence Interval) among pregnant, lactating and reproductive-age women

**Nutrients**	**EAR (mg)**	Mean (mg)^*^	**95% CI**	**% inadequacy**	**95% CI**
**Vitamin A**					
Pregnant women	550	471.6	390.4; 552.7	71.0	61.2; 80.8
Lactating women	900	398.4	353.3; 443.5	95.0	93.0; 96.9
Reproductive-age women	500	415.7	393.4; 437.4	72.0	70.0; 73.9
**Vitamin C**					
Pregnant women	70	133.1	100.4; 165.8	40.0	28.2; 51.7
Lactating women	100	127.5	106.1; 148.9	56.0	48.1; 63.8
Reproductive-age women	60	126.4	118.7; 134.0	37.0	35.0; 38.9
**Vitamin B6**					
Pregnant women	1.6	1.5	1.3; 1.7	59.0	43.3; 74.6
Lactating women	1.7	1.4	1.4;1.4	75.0	71.0; 78.9
Reproductive-age women	1.1	1.4	1.4; 1.4	33.0	31.0; 34.9
**Vitamin B12**					
Pregnant women	2.2	5.7	4.1; 7.2	6.0	1.8; 13.8
Lactating women	2.4	4.5	3.5; 5.5	19.0	7.2; 30.7
Reproductive-age women	2.0	4.3	4.1; 4.5	12.0	10.0; 13.9
**Calcium**					
Pregnant women	800	573.3	421.9; 724.6	82.0	68.2; 95.7
Lactating women	800	461.2	431.6; 490.8	92.0	90.0; 93.9
Reproductive-age women	800	477.3	467.9; 486.7	91.0	89.9; 92.9
**Iron**					
Pregnant women	22	11.2	9.2; 13.2	97.0	-
Lactating Women	6.5	10.5	9.7; 11.3	15.7	-
Reproductive-age women	8.1	10.3	10.1; 10.5	28.0	-
**Zinc**					
Pregnant women	9.5	11.6	9.2; 13.9	35.0	11.4; 58.5
Lactating women	10.4	9.6	7.6; 11.2	64.0	44.4; 83.6
Reproductive-age women	6.8	10.1	9.9; 10.3	20.0	20.0; 20.0
**Folate**					
Pregnant women	520	405.1	364.7; 445.5	78.0	70,2; 85.8
Lactating women	450	379.1	357.5; 400.7	72.0	66.1; 77.9
Reproductive-age women	320	375.2	358.5; 391.9	40.0	36.0; 43.9

EAR- Estimated Average Requirement.

*Except for Vitamin A (μg RAE) and Folate (mcg DFE).

The nutrient with the highest inadequate intake in pregnant women was iron (97%); in lactating women, vitamin A (95%); and in reproductive-age women was calcium (91%). The intake of calcium was also very inadequate in lactating and pregnant women.

Both pregnant and reproductive-age women reported an inadequate intake greater than 70% for vitamin A and folate and lower than 15% for vitamin B12. The prevalence of inadequate intake of vitamin C was highest in the group of lactating women (56%), followed by pregnant women (40%) and reproductive-age women (37%). The percentage of sodium intake above the tolerable maximum value (Table 4) was greater than 70% among all women studied.

**Table 4:** UL, average intake and% above UL(with their respective 95% Confidence Interval) of sodium among pregnant, lactating, and reproductive-age women

**Sodium**	**UL (g)**	**Mean (g)**	**95% CI**	**% above UL**	**95% CI**
Pregnant women	2.3	3.1	2.8; 3.4	79.0	71.1; 86.8
Lactating women	2.3	2.8	2.5; 3.2	70.0	58.2; 81.7
Reproductive-age women	2.3	2.8	2.8; 2.9	70.0	68.0; 71.9

UL- Tolerable Upper Intake Level.

## Discussion

In this study we found that pregnant and lactating women do not adopt a food consumption pattern that enables them reach their nutritional goals.

For most nutrients, recommendations are greater for pregnant and lactating women, increasing the prevalence of inadequate nutrient intake. A way to increase their nutrient intake would be by changing the proportion of food sources of nutrients in the diet, i.e., giving preference to nutrient-rich foods. However, there are no specific recommendations for either pregnant or lactating women to choose different foods from those that comprise a healthy diet for general women, and it appears that there is no scientific evidence for that. Thus, the increase in nutrient intake by pregnant and lactating women is expected to be due to the increase in food amount and consequently total energy of the diet, but in this study, we did not find higher intakes of nutrients and energy.

A concern with weight gain could be an explanation for these findings [25]. The prevalence of women who are either overweight or obese is high in Brazil, reaching 48% and 16.9% respectively in this present survey. Parity is an important risk factor for obesity in Brazil [26] and elsewhere [27,28], but we have no data on dietary restriction among pregnant and lactating women in Brazil.

In addition, the recommendation to increase the energy intake during pregnancy applies only to second and third trimesters [19]. Thus, it is difficult for pregnant women who are in the first trimester to achieve their nutrient intake recommendations, which are the same for the entire pregnancy, without an increase in food intake. We have no information about gestational age of pregnant women in our study. Assuming an equal proportion of them through the three trimesters of pregnancy, it is expected that approximately one-third of them (which probably are in the first trimester) would not reach their nutrient recommendation as their food intake would probably be similar to preconception.

Most of the cut offs to evaluate nutrient inadequacies are based on serum concentration and body storage that are necessary to a specific metabolic task. Although inadequate nutrient intake is common in both developed and undeveloped countries, there are few reports of clinical signs of these insufficient intakes. The exception is for two key nutrients that receive special attention in public health: iron and folate. Approximately 75% of all anaemia diagnosed during pregnancy is due to iron deficiency [29], and the prevalence of iron-deficiency anaemia among pregnant women is 29.1% in Brazil [30]. Brazil has mandatory flour fortification with iron and folic acid and our analysis accounted for iron and acid folic from fortification products, but the inadequacy of these nutrients was still high.

According to our results, it is unlikely that the iron requirements of most pregnant women in Brazil can be met through the diet alone, even with iron fortification. Brazilian Ministry of Health launched in 2005, the National Iron Supplementation Program, which promotes iron supplementation in pregnant women from 20 weeks of gestational until the third month postpartum. Pregnant women receive ferrous sulphate (60 mg) and folic acid (5 mg) to be administered every day [31]. Although this program provides nearly three times the EAR of iron (22 mg) [19], the prevalence of anaemia is still high in this group. The low adherence to supplementation programs as a result of side effects (diarrhea, constipation, stomach discomfort, heartburn and nausea) [32] and the low absorption of iron after taking these drugs (approximately 11, 7%) [33] explain the high prevalence of anaemia in pregnant women. Thus, this group is placed at even higher risk of iron deficiency, and the necessity of increasing the iron density in their diet is emphasised.

The very high prevalence of inadequate intake of vitamin A and calcium and an excessive intake of sodium was observed in the overall population, indicating a need for changes in the dietary pattern of Brazilians. According to the Household Budget Survey 2008/2009, the intake of processed foods high in energy and low in nutrients increased at the expense of healthy foods that are rich in nutrients such as grains, beans, fruits and vegetables, which were traditionally part of the diet in Brazil. This may in part explain the high prevalence of inadequate nutrient intake in Brazil.

The Brazilian Program for Humanization of Prenatal and Childbirth Care, launched in 2000 [34], offers prenatal assistance to pregnant women and gives them information about infant care, breastfeeding, balanced diet and physical exercise. A recent report showed that the goal of six visits during the pregnancy [35] was reached by 80.9% of pregnant women [36]. Despite the increasing coverage and access of prenatal care in Brazil [37], our results indicate that nutritional guidelines are ineffective, because there is no apparent adaptation to the higher nutrient requirements in the diet of pregnant woman.

Usual nutrient and food intakes were estimated using recent recommended models to predict intake and remove within-person variation. Neglecting this leads to serious error in the results, underestimating higher prevalence and overestimating lower prevalence of inadequate intakes. The NCI model [12,17] estimates mean and percentiles of usual nutrient and food intakes and it is suitable to account for complex sample design.

This was the first time that the food consumption of pregnant and lactating women was extensively investigated in Brazil. The limitations of this study are related to the cross-sectional design, which does not allow the investigation of changes in the same women, before and during pregnancy and lactation and the limitations are the same as those of any study based on reported data on consumption, in particular, underreporting of intake. Accuracy in reporting intake may vary greatly between respondents depending on factors such as education, socio-economic status and other respondent characteristics. Also, respondent characteristics may be associated with either inaccurate reporting in diaries or with changing eating patterns during recording periods.

In another study using all participants of NDS, it was estimated an underreporting of energy consumption of about 17% occurred, individuals with normal weight showed underreporting of approximately 13% [11]. These results are consistent with those obtained in other reviews of dietary records against doubly labeled water held in Brazil [38,39]. Volunteers with a higher body fat percentage tended to under-report more [38].

It has been found in individuals with underreporting of energy intake, lower densities of carbohydrates, sugar, alcohol and saturated fatty acids and higher densities of protein, starch, fiber, mono and polyunsaturated fatty acids and cholesterol [40,41]. There is no reason to believe that underreporting could be different between the three groups of women evaluated.

## Conclusion

In conclusion, pregnant and lactating women in Brazil do not adequately change their food intake in accordance with their needs, and all women, regardless of their reproductive cycle, have a diet of low nutrient density. Dietary counseling of women should be initiated even before pregnancy, because according to the results, most reproductive-age women have inadequate intakes of various nutrients. A more intensive nutritional education program is fundamental to change dietary patterns as women make the transition into pregnancy and then lactation. Strong dietary counseling of women at this time of the life cycle may improve the overall diet of the family, a necessary step to prevent and correct nutritional deficiencies.

## Competing interests

The authors declare that they have no competing interests.

## Authors’ contributions

The authors’ contributions are as follows: SQ and V-JE performed the statistical analyses, interpreted the data, and wrote the paper; SR and MDML performed the interpretation of the data and wrote the paper. All authors actively participated in the manuscript preparation, and they all read and approved the final version of the manuscript.

## Pre-publication history

The pre-publication history for this paper can be accessed here:

http://www.biomedcentral.com/1471-2393/14/186/prepub
